# The National Medical Union

**Published:** 1914-01-24

**Authors:** Robert Carswell, E. Arthur Dorrell

**Affiliations:** Hon. Secretary; Hon. Secretary


### The National Medical Union.

A meeting of the Executive Committee was held at the offices of the Union, 346 Strand, on Saturday, January 17. Present: Professor Russell (President), in the chair, Drs. Greenyer, Oppenheimer, Heatherley, Spurgin, Lowe, Greene, Hemmans, O'Connor, Pinder, Sharman, Johnston, Carswell, and Mr. Dorrell.

A letter was read from Dr. O. R. Lewis, of Birmingham, asking whether there was anything in the rules of the Union forbidding the signing of Form 43 IC. The Executive Committee, after considering the matter, decided to make it the subject of a recommendation to the Council.

In connection with a letter from Charles J. Cooke, of Plymouth, asking for the views of the National Medical Union upon the Seamen's National Insurance Society, it was also decided to refer to the Council the consideration of the eligibility of medical officers of the Seamen's Society for membership of the Union.

The memorandum of policy formulated by a sub-committee appointed for the purpose, and published on January 2, was read, commented upon by Dr. Greenyer, and formally approved by the committee.

The subjects of organisation and constitution were next considered, and it was resolved: (1) That it be a recommendation to the Council to proceed to the formation of an Organisation Committee for the purpose of taking the necessary steps to enrol all non-panel members of the profession who are in sympathy with the objects and policy of the Union; (2) that the Council be recommended to appoint a Constitution Committee to consider and report upon proposals for a constitution for the Union.

It was recommended that the Constitution Committee should be a small one, consisting of the following members, Drs. Oppenheimer, O'Connor, and Edwin Smith, all of whom are members of the Bar, together with the honorary secretaries.

At the conclusion of the meeting Dr. Heatherley laid before the committee a detailed account of the energetic action which the Liverpool Non-Panel Union was taking in its district.

A hearty vote of thanks was accorded to Professor Russell, who had come from Edinburgh for the special purpose of attending this meeting of committee.

There will be a meeting of the Provisional Federating Council on Saturday, January 31, at 4.30 P.M., at the offices of the Union.

Great interest and importance are attached to the presence of Professor Russell at Saturday's meeting. Those who have a knowledge of the stand made by the profession in Scotland against the Act will remember that Professor Russell was Chairman of the Scottish National Insurance Council. This Council was composed of representatives of the four Scottish Universities, the three medical corporations, and other bodies, and was the most generally representative of all the organisations formed at that time to safeguard the interests of the profession.

At its first meeting, early in 1912, the Council placed on record its complete sympathy with the widespread dissatisfaction and regret that the profession should not have been adequately consulted; it affirmed its adherence to "the six cardinal points"; and it was prepared, in the event of these not being granted, to formulate a public medical service of its own.

In December 1912 a circular issued by the Colleges and the Faculty affirmed an earnest desire "to help the profession to maintain its independence and to assist it in endeavouring to obtain such modifications of the medical provisions of the Act as would make these consistent with the best traditions and ideals of the profession." The Colleges and Faculty stated that "they could not be parties to an agreement which was derogatory to the status of the medical profession; that they were assured that many practitioners believed, with them, that this was still the case; and that they wished it to be understood that the profession in adhering to that view might count upon their sympathy and support."

The National Medical Union is keenly alive to the importance attached to the sympathy and support of the leaders of the profession, and deeply appreciates Dr. Russell's acceptance of office as one of the Presidents of the Union and his active participation in the work of organisation.

Robert Carswell,
E. Arthur Dorrell,
Hon. Secretaries.

